# The Impact of Sleep‐Related Disorders on the Sleep Quality in Patients With Multiple Sclerosis in a Saudi Cohort

**DOI:** 10.1155/nri/4041733

**Published:** 2026-07-08

**Authors:** Ismail A. Khatri, Khalid Bin Aziz, Esraa Y. Arabi, Ghaida A. Almusallam, Razan F. Alfaiz, Abeer A. Alkhathlan, Nazish Masud, Yaser Al Malik

**Affiliations:** ^1^ Department of Neurology, King Abdulaziz Medical City, Ministry of National Guard Health Affairs, Riyadh, Saudi Arabia, ngha.med.sa; ^2^ College of Medicine, King Saud bin Abdulaziz University for Health Sciences, Riyadh, Saudi Arabia, ksau-hs.edu.sa; ^3^ King Abdullah International Medical Research Center, Riyadh, Saudi Arabia, kaimrc.med.sa; ^4^ Department of Surgery, King Abdulaziz Medical City, Ministry of National Guard Health Affairs, Riyadh, Saudi Arabia, ngha.med.sa; ^5^ Department of Internal Medicine, King Saud Medical City, Riyadh First Health Cluster, Ministry of Health, Riyadh, Saudi Arabia, moh.gov.sa; ^6^ King Abdullah bin Abdulaziz University Hospital, Princess Nourah bint Abdulrahman University, Riyadh, Saudi Arabia, pnu.edu.sa; ^7^ Department of Family Medicine, Riyadh Second Health Cluster, Ministry of Health, Riyadh, Saudi Arabia, moh.gov.sa; ^8^ Department of Biostatistics, Epidemiology, and Environmental Health Sciences, Jiann-Ping Hsu College of Public Health, Georgia Southern University, Statesboro, Georgia, USA, georgiasouthern.edu

**Keywords:** disease-modifying therapy, EDSS, Kingdom of Saudi Arabia, multiple sclerosis, quality of sleep, sleep disorders

## Abstract

**Background:**

Multiple sclerosis (MS) causes physical as well as psychosocial disability in young adults. Various sleep‐related disorders have been reported in patients with multiple sclerosis (pwMS).

**Objectives:**

We determined the frequency and patterns of various sleep‐related disorders in pwMS and correlated those with the sleep quality.

**Methods:**

This was an IRB‐approved study done at King Abdulaziz Medical City, MNGHA, Riyadh, KSA. Informed consent was obtained. Validated questionnaires including Pittsburgh Sleep Quality Index (PSQI), Sleep Disorders Questionnaire (SDQ), and STOP‐BANG Questionnaire were used. Data were analyzed using SPSS.

**Results:**

A total of 169 pwMS were included; 116 (69%) were females. Mean age was 35 ± 10 years; mean BMI was 27.6 ± 16.8 kg/m^2^. Almost half (47%) of the patients were employed, and most worked in day shift (85%). Caffeinated beverages were used by 87%; 16% were smokers. Relapsing remitting MS was the commonest type in 93%; 82% were using disease‐modifying therapy (DMT). Sleep‐related medications were used by 18%. Disability measured by EDSS was mild in 64%, moderate in 18%, and severe in 18%. Poor quality of sleep was reported by 74% (mean PSQI 7.8 ± 3.9). Circadian rhythm disorders were reported in 22%, psychiatric disorders in 18%, and parasomnias in 14%; 4% had high risk for sleep apnea. Psychiatric disorders (*p* = 0.05) and movement disorders (*p* = 0.05) were more frequently reported in women. Risk for insomnia (*p* = 0.03), circadian rhythm disorders (*p* = 0.013), and psychiatric disorders (*p* = 0.002) were associated with poor sleep quality. Type of MS and current treatment did not affect the frequency of sleep disorders or sleep quality.

**Conclusions:**

In our cohort of pwMS, sleep‐related disorders were common. Risk for insomnia, psychiatric disorders, and circadian rhythm disorders were associated with poor sleep quality. Certain disorders showed a trend toward female preponderance. Disease severity and use of DMTs did not affect sleep quality in our cohort.

## 1. Introduction

Multiple sclerosis (MS) is frequently accompanied by a broad spectrum of sleep‐related disorders that can profoundly affect patient well‐being. Recent studies indicate that approximately half of patients with MS (pwMS) experience chronic sleep disturbances or poor sleep quality, a prevalence much higher than in the general population [**1**]. Insomnia is one of the most common sleep‐related disorders in MS, with about 50% of patients reporting significant insomnia symptoms and roughly 20% meeting criteria for an insomnia disorder [**1**]. Other sleep‐related disorders are also over‐represented in pwMS. Restless legs syndrome (RLS), characterized by an irresistible urge to move the legs at night, has a pooled prevalence of about 28% in MS (versus ∼8% in the general population) [2]. Likewise, obstructive sleep apnea (OSA) is found in approximately 25%–36% of pwMS (depending on screening method) [3]. Even less common conditions such as rapid eye movement (REM) sleep behavior disorder and narcolepsy, while affecting only a minority of pwMS, have been documented at greater frequencies than in healthy individuals [4]. These disturbances often manifest clinically as prolonged sleep latency, fragmented nocturnal sleep, excessive daytime sleepiness, often resulting in chronic fatigue, and poor quality of life (QoL) in pwMS.

The pathophysiology underlying the link between MS and sleep disturbances is multifactorial. MS‐related demyelinating lesions can directly damage key neuroanatomical regions that regulate sleep–wake cycle including hypothalamus and brainstem [5]. Concurrently, multiple secondary factors common in MS that contribute to poor sleep quality, including painful spasms, neuropathic pain, nocturnal muscle cramps, and urinary frequency due to neurogenic bladder, can all cause frequent awakenings. Additionally, side effects of certain MS treatments (for instance, corticosteroids or stimulants) and comorbid conditions (such as depression or iron deficiency) further disturb sleep continuity and quality [4]. The convergence of these direct neurological insults and indirect factors results in a high burden of sleep‐related disorders among pwMS.

Recognizing and addressing sleep‐related disorders in MS is critical, as these disorders can significantly impact the disease course and patients’ QoL. Sleep disturbances in MS are not merely benign bystanders but are associated with worse clinical outcomes. Fatigue, one of the most debilitating MS symptoms, is often exacerbated by coexisting sleep‐related disorders; studies have shown that pwMS with conditions like insomnia, RLS, or OSA tend to report higher fatigue levels [6]. Likewise, cognitive functions can be affected in these individuals; poor sleep quality and untreated sleep‐related disorders have been linked to slower information processing and memory deficits in MS [**4**]. Chronic insomnia and RLS correlate with higher rates of anxiety and depression in MS populations [**6**]. Critically, impaired sleep has a measurable negative effect on overall QoL in MS. In a large cohort study, 67% of pwMS had objectively poor sleep quality, which was independently associated with substantially lower health‐related QoL scores [**7**]. Importantly, emerging evidence suggests that effective management of sleep‐related disorders may improve outcomes in pwMS. Treatment of conditions such as RLS or OSA in MS has been observed to alleviate fatigue and daytime dysfunction and to potentially enhance cognitive performance and mood [**4**]. Despite the high prevalence and clinical importance of sleep disturbances in MS, these issues often remain under‐recognized in routine practice, with attention traditionally focused on the motor and immunological aspects of the disease.

Much of the existing literature on MS‐related sleep disorders is derived from North American, European, or Australian populations, and data from Saudi Arabia and the Middle East are relatively scarce. Cultural, lifestyle, and genetic factors could influence the presentation of sleep‐related disorders in MS, highlighting the need for region‐specific research. Notably, a recent pilot study in Saudi Arabia found RLS in nearly one‐third of pwMS and was associated with greater disability, heightened fatigue, and worse mental health scores, highlighting the potential impact of sleep‐related disorders in the local MS population [**6**]. Yet, aside from this single‐disorder study, there remains a lack of comprehensive data on the full spectrum of sleep‐related disorders in Saudi Arabia and Middle Eastern MS cohorts. To address this knowledge gap, the present study was conducted to assess the frequency and patterns of sleep‐related disorders in pwMS at King Abdulaziz Medical City (KAMC), Ministry of National Guard Health Affairs (MNGHA) in Riyadh, Kingdom of Saudi Arabia (KSA). By characterizing the prevalence and clinical correlates of sleep‐related disorders in pwMS, we aimed to deepen the understanding of MS‐related sleep issues and to potentially suggest targeted interventions that could improve the overall disease management and QoL of pwMS, particularly in Saudi Arabia and Middle Eastern setting.

## 2. Materials and Methods

### 2.1. Study Design and Setting

This study was a cross‐sectional survey conducted in the MS and general neurology clinics at King Abdulaziz Medical City, MNGHA, Riyadh, KSA. The study was approved by the institutional review board (IRB) of King Abdullah International Medical Research Center (KAIMRC) (approval number RC19/099/R) prior to COVID‐19 outbreak; however, due to severe restrictions and closure of physical visits to the clinics during COVID‐19 epidemic, data collection was delayed. Based on an expected prevalence of sleep‐related disorders of 50% in MS patients, with a 95% confidence level and 5% margin of error, the required sample size was estimated to be 169 participants.

### 2.2. Participants

Eligible participants were adults aged ≥ 18 years with a confirmed clinical diagnosis of MS (any subtype). The diagnosis of MS was made on the basis of McDonald’s criteria, revision 2017, which included spinal cord lesions. We excluded patients with conditions that could confound sleep assessment or impede participation, including neurological comorbidity that could affect sleep, for example, narcolepsy, pre‐existing dementia or other neurodegenerative diseases, or refusal to consent or inability to complete the questionnaires. Patients with any other medical condition that could potentially cause sleep disturbance, for example, severe chronic pulmonary disease or congestive heart failure, were also excluded. Consecutive pwMS meeting inclusion criteria were invited to participate during routine clinic visits in nonrandom fashion. No monetary or therapeutic incentives were provided. Informed consent was obtained on IRB‐approved form before data collection.

### 2.3. Data Collection and Measurement Tools

A structured data collection form was used to collect all data. Demographic information (age, sex) and clinical characteristics were obtained through patient interview and electronic medical records review. Disease‐specific data, including MS subtype classification, categorized as relapsing‐remitting MS (RRMS), primary‐progressive MS (PPMS), secondary‐progressive MS (SPMS), or progressive‐relapsing MS (PRMS), as well as current use of any disease‐modifying therapies (DMTs) for MS, were obtained from electronic medical records. The severity of MS disability was assessed for each patient using the Expanded Disability Status Scale (EDSS), recorded as the most recent EDSS score documented in the clinic. We also recorded whether the patient was currently using any prescription sleep medications.

Sleep‐related disorders were evaluated with validated self‐report questionnaires, without additional objective testing. Participants completed the Pittsburgh Sleep Quality Index (PSQI), a 19‐item questionnaire that assesses overall sleep quality and disturbances over a 1‐month interval [**8**]. The PSQI provides a global score (range 0–21), with higher scores indicating poorer sleep quality. We used the PSQI to quantify subjective sleep quality in our cohort. A score of > 5 indicated poor sleep quality. The Sleep Disorders Questionnaire (SDQ), a comprehensive instrument that evaluates symptoms of various primary sleep‐related disorders, was also used [**9**]. The SDQ was used as a differential diagnostic tool, yielding profile scores for different sleep‐related disorder categories. The SDQ is composed of 16 questions. Each question can be answered in five different grades, including never, rarely, occasionally, most night/days, and always. The questions are so arranged that questions 1–5 assess the possibility or risk of insomnia, questions 6–9 assess the possibility of presence of psychiatric disorders, question 10 assesses the possibility of circadian rhythm disorder, questions 11–12 inquire about the possibility of movement disorders, question 13 inquires about insomnia, and questions 14–16 screen for features of OSA. There are standard guidelines and scoring system to analyze the answers to these questions. We used the general guidelines for the interpretation of SDQ and used the scoring system accordingly. Furthermore, the STOP‐BANG Questionnaire, which is an eight‐item screening tool for OSA risk (addressing Snoring, daytime Tiredness, Observed apnea, high blood Pressure, BMI, Age, Neck circumference, and Gender), was administered to estimate each patient’s risk of OSA [**10**]. A higher STOP‐BANG score (range 0–8) indicates greater likelihood of clinically significant sleep apnea; however, it does not confirm the diagnosis of OSA. We did not perform formal sleep studies to confirm the presence of OSA, or any other sleep‐related disorder clinically. All the results presented are based on questionnaires with no confirmatory clinical or laboratory diagnosis. When available, the Arabic translations of the instruments were used. If not available, the instruments were translated into Arabic and back‐translated into English.

No formal clinical sleep diagnoses were made in this study; instead, the presence of sleep‐related disorders was determined solely based on the above questionnaire results. For analysis, each patient’s sleep‐related disorder status was classified into one or more of the following categories (as indicated by their questionnaire scores): positive screening for insomnia, psychiatric sleep‐related disorder, circadian rhythm sleep–wake disorder, parasomnia, movement disorder, and sleep apnea. These categories correspond to the primary domains evaluated by the SDQ and STOP‐BANG tools. Patients could fall into multiple categories if they had indications of more than one type of sleep‐related disorder.

### 2.4. Statistical Analysis

All data were analyzed using IBM SPSS Statistics software (IBM Corp., Armonk, NY, USA). Continuous variables (e.g., age, EDSS score, questionnaire scores) were summarized as mean ± standard deviation (SD) or median with interquartile range (IQR), as appropriate. Categorical variables (e.g., sex, MS subtype, presence of specific sleep‐related disorder) were described as frequencies and percentages. Comparisons between groups were conducted using chi‐square or Fisher’s exact tests for categorical variables, and independent‐sample *t*‐tests for continuous variables. Associations between sleep quality (good vs. poor) and specific sleep‐related disorder categories were evaluated using these univariate comparisons. A *p* value ≤ 0.05 was considered statistically significant. Missing data were not imputed, and only complete cases were included in the analyses.

### 2.5. Ethical Considerations

The study was conducted in accordance with the ethical standards after approval by the local IRB. Informed consent was obtained from all participants prior to enrollment.

## 3. Results

In total, 169 patients with MS were included in the study. The cohort was predominantly female, 116 (69%) with a mean age of 35 ± 10 years. The mean body mass index (BMI) was 27.6 ± 16.8 kg/m^2^ (range 22–29; median IQR 25.8), and the mean neck circumference was 35.1 ± 3.8 cm. The majority of patients were well educated, 143 (85%) had at least a high school education and 98 (58%) were married. Nearly half of the cohort 79 (47%) were employed (predominantly day‐shift workers), and 147 (87%) reported regular daily use of caffeinated beverages. Twenty‐seven (16%) of pwMS were current smokers, consuming a median of 20 cigarettes per day. Relapsing‐remitting MS (RRMS) was the most common disease subtype, accounting for 157 (93%) of patients, with only 7% having progressive forms of MS. Most pwMS 139 (82%) were receiving DMT for MS at the time of interview, and disability levels were generally low: 108 (64%) had mild disability (EDSS 0–3), while 31 (18%) and 30 (18%) each had moderate (EDSS 3.5–5.5) or severe disability (EDSS ≥ 6), respectively. A minority of pwMS 30 (18%) reported using sleep medications, and 15 (9%) had a family history of psychiatric disorders. Baseline demographic and clinical features of the MS patients are summarized in Table 1.

**TABLE 1:** Demographic and clinical characteristics of the patients with MS cohort (*N* = 169).

Variable	Category/statistic	Value
Age (years)	Mean ± SD	35 ± 10
	Median (Q1–Q3)	34 (28–41)

Height (cm)	Mean ± SD	162.8 ± 10.5
	Median (Q1–Q3)	162 (157–169)

Weight (kg)	Mean ± SD	71.1 ± 19.7
	Median (Q1–Q3)	67 (58–80)

Neck circumference (cm)	Mean ± SD	35.1 ± 3.8
	Median (Q1–Q3)	35 (32–37)

Body mass index (BMI, kg/m^2^)	Mean ± SD	27.56 ± 16.76
	Median (Q1–Q3)	25.8 (22–29)

BMI category	Underweight (< 18.5)	7 (4%)
	Normal (18.5–24.9)	68 (40%)
	Overweight (25–29.9)	56 (33%)
	Obese (≥ 30)	38 (23%)

Sex	Male	53 (31%)
	Female	116 (69%)

Marital status	Single	71 (42%)
	Married	98 (58%)

Education level	No formal education	2 (1%)
	Elementary/middle school	11 (7%)
	High school	58 (34%)
	Bachelor’s degree	72 (43%)
	Master’s/PhD	13 (8%)
	Other	13 (8%)

Monthly household income	< 10,000 SAR	55 (33%)
	11–20,000 SAR	59 (35%)
	> 20,000 SAR	35 (21%)
	Not reported	20 (12%)

Employment status	Employed	79 (47%)
	Unemployed	90 (53%)

Work shift (if employed)	Day shift	68 (85%)
	Evening shift	2 (3%)
	Rotating (day + night)	10 (13%)

Regular caffeine use	Yes	147 (87%)

Type of caffeine (among users)	Tea	53 (31%)
	Coffee	73 (43%)
	Arabian coffee	99 (59%)

Smoking status	Smoker	27 (16%)
	Nonsmoker	142 (84%)

Cigarettes per day	Mean ± SD (among smokers)	22 ± 19
	Median (Q1–Q3)	20 (10–21)

On sleep medication	Yes	30 (18%)

Family history of psychiatric disorder	Yes	15 (9%)

MS subtype	Relapsing‐remitting (RRMS)	157 (93%)
	Primary‐progressive (PPMS)	5 (3%)
	Secondary‐progressive (SPMS)	7 (4%)
	Progressive‐relapsing (PRMS)	0 (0%)

On disease‐modifying therapy	Yes (current DMA use)	139 (82%)

MS disability status (EDSS)	Mild (0–3)	108 (64%)
	Moderate (3.5–5.5)	31 (18%)
	Severe (≥ 6)	30 (18%)

*Note:* Percentages may not total 100% within some rows due to rounding; Q1–Q3 = first and third quartiles (interquartile range); EDSS = Expanded Disability Status Scale (higher scores indicate greater disability).

Abbreviations: BMI = body mass index, DMA = disease‐modifying agent, MS = multiple sclerosis, PPMS = primary‐progressive MS, PRMS = progressive‐relapsing MS, RRMS = relapsing‐remitting MS, SAR = Saudi Arabian Riyals, SD = standard deviation, SPMS = secondary‐progressive MS.

### 3.1. Prevalence of Sleep‐Related Disorders and Sleep Quality

**FIGURE 1:**
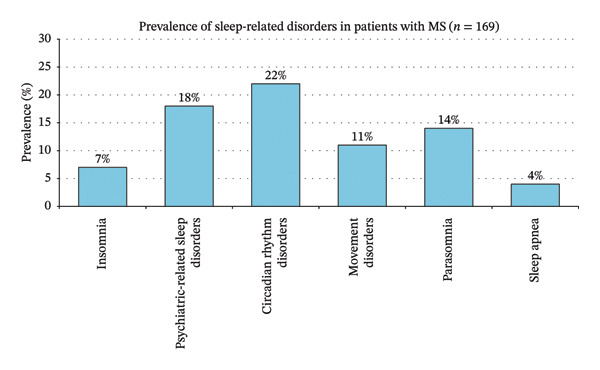
Prevalence of specific sleep‐related disorder categories among pwMS. This figure illustrates the proportion of the study cohort that screened positive for each type of sleep‐related disorder (*Note*. Categories are not mutually exclusive, as some patients had indications of multiple sleep disorders).

Overall, 125 (74%) of patients were classified as having poor sleep quality based on their global PSQI scores. In terms of specific sleep‐related disorders, a substantial proportion of patients screened positive for at least one disorder category (Figure 1). The most frequently reported sleep‐related disorders were circadian rhythm sleep–wake disorders, present in 38 (22%), and psychiatric sleep‐related disorders, reported in 30 (18%). Symptoms of parasomnias (such as nightmares or possible REM sleep behavior disorder symptoms) were reported in 24 (14%) of patients. Symptoms of movement disorders, a category primarily reflecting RLS or periodic limb movement symptoms, were present in 18 (11%). Positive screening for insomnia was noted in 12 (7%) patients. Risk of OSA risk was the least common, with only 7 (4%) patients having a high‐risk STOP‐BANG score for OSA. The presence of OSA was not confirmed with any objective laboratory testing, hence a definitive diagnosis of OSA could not be made. These results are illustrated in Figure 1.

### 3.2. Associations With Sleep Quality

We next assessed whether the presence of specific sleep‐related disorders was associated with worse sleep quality in our pwMS. Patients who screened positive for insomnia, circadian rhythm disturbance, or a psychiatric sleep‐related disorder were significantly more likely to report poor sleep quality. Notably, 100% of patients who screened positive for insomnia had poor sleep quality (PSQI > 5), compared to 72% of those without insomnia symptoms (*p* = 0.03). Similarly, 97% of patients with a psychiatric sleep‐related disorder had poor sleep quality, versus 69% without (*p* = 0.002). Nearly 90% of patients with features of circadian rhythm sleep–wake disorders reported poor sleep quality, compared to about 70% of those without such a disorder (*p* = 0.013). By contrast, the proportions of poor sleepers did not differ significantly by the presence vs. absence of symptoms of movement disorders (89% vs 72% with poor sleep; *p* = 0.16), parasomnias (88% vs 72%; *p* = 0.13), or high risk for OSA (71% vs 74%; *p* ≈ 0.7). These data are summarized in Table 2.

**TABLE 2:** Relationship between presence of specific sleep‐related disorders and sleep quality (good vs poor) in pwMS (*N* = 169).

Sleep‐related disorder	Status	Patients, *n* (% total)	Good sleep, *n* (% status)	Poor sleep, *n* (% status)	*p* value
Insomnia	Absent	157 (93%)	44 (28%)	113 (72%)	0.03
	Present	12 (7%)	0 (0%)	12 (100%)	

Psychiatric‐related sleep disorder	Absent	139 (82%)	43 (31%)	96 (69%)	0.002
	Present	30 (18%)	1 (3%)	29 (97%)	

Circadian rhythm sleep–wake disorder	Absent	131 (78%)	40 (31%)	91 (70%)	0.013
	Present	38 (22%)	4 (11%)	34 (89%)	

Movement disorder (e.g. RLS)	Absent	151 (89%)	42 (28%)	109 (72%)	0.16
	Present	18 (11%)	2 (11%)	16 (89%)	

Parasomnia	Absent	145 (86%)	41 (28%)	104 (72%)	0.13
	Present	24 (14%)	3 (13%)	21 (88%)	

Obstructive sleep apnea (OSA) risk	Absent	162 (96%)	42 (26%)	120 (74%)	0.71
	Present	7 (4%)	2 (29%)	5 (71%)	

*Note:* Percentages reflect the proportion of patients with good or poor sleep within each disorder status category. Percentages may not total 100% within some rows due to rounding.

Abbreviations: OSA = obstructive sleep apnea, RLS = restless legs syndrome.

Consistent with the above findings, global PSQI scores were significantly higher (worse sleep quality) in patients who had most types of sleep‐related disorders (Table 3). Patients who screened positive for insomnia had a mean PSQI score of 14 ± 2, compared to 7 ± 4 in those without insomnia (*p* < 0.001). Those with a psychiatric sleep‐related disorder had an average PSQI of 12 ± 3 vs 7 ± 3 in those without (*p* < 0.001), and patients with a circadian rhythm disorder had a mean PSQI of 11 ± 4 vs. 7 ± 3 in those without (*p* < 0.001). Parasomnia and movement disorder symptoms were also associated with moderately higher PSQI scores: the presence of symptoms of movement disorder corresponded to a mean PSQI of 10 ± 4 vs 8 ± 4 (*p* = 0.02), and the presence of a parasomnia was associated with a PSQI of 10 ± 4 vs 7 ± 4 (*p* < 0.001). In contrast, patients at high risk for OSA did not have significantly different PSQI scores compared to those not at risk (10 ± 6 vs 8 ± 4, *p* = 0.27). These results indicate that subjective insomnia, circadian rhythm disturbances, and psychiatric sleep‐related disorders in particular were linked to substantially worse subjective sleep quality in our pwMS.

**TABLE 3:** Comparison of global PSQI scores in patients with or without specific sleep‐related disorders (*N* = 169).

Sleep‐related disorder	Absent (*n*, %)	Present (*n*, %)	PSQI mean ± SD (absent)	PSQI mean ± SD (present)	*p* value
Insomnia	157 (93%)	12 (7%)	7 ± 4	14 ± 2	< 0.001
Psychiatric‐related sleep disorder	139 (82%)	30 (18%)	7 ± 3	12 ± 3	< 0.001
Circadian rhythm sleep–wake disorder	131 (78%)	38 (22%)	7 ± 3	11 ± 4	< 0.001
Movement disorder (RLS/PLMS)	151 (89%)	18 (11%)	8 ± 4	10 ± 4	0.02
Parasomnia	145 (86%)	24 (14%)	7 ± 4	10 ± 4	< 0.001
Obstructive sleep apnea (OSA) risk	162 (96%)	7 (4%)	8 ± 4	10 ± 6	0.27

Abbreviations: OSA = obstructive sleep apnea, PLMS = periodic limb movements of sleep, RLS = restless legs syndrome.

When stratified by sex and disability level, most sleep‐related disorder categories showed no significant group differences. Female patients showed a trend of higher prevalence of psychiatric sleep‐related disorders and movement disorder‐type sleep disturbances compared to male patients: 25 (22%) vs. 5 (9%) and 16 (14%) vs. 2 (4%), respectively; *p* = 0.05 for both. In contrast, the proportions of patients who screened positive for insomnia, circadian rhythm sleep–wake disorders, parasomnias, and poor sleep quality did not differ significantly between females and males: 9 (8%) vs. 3 (6%), 27 (23%) vs. 11 (21%), 17 (15%) vs. 7 (13%), and 86 (74%) vs. 39 (74%), respectively; all *p* > 0.70. Similarly, disease‐related disability (EDSS strata) was not associated with significant differences in most sleep‐related disorders or overall sleep quality. Poor sleep quality was highly prevalent across mild, moderate, and severe disability groups: 78 (72%), 26 (84%), and 21 (71%), respectively; *p* = 0.36. The only notable association was observed for movement disorders, which increased in frequency with greater disability: 8 (7%) in EDSS 0–3 vs. 3 (10%) in EDSS 3.5–5.5 and 7 (23%) in EDSS ≥ 6; *p* = 0.05. These borderline differences may be due to the small number of pwMS in each subgroup. No other statistically significant differences were found across EDSS categories for subjective insomnia (*p* = 0.83), psychiatric sleep‐related disorders (*p* = 0.94), circadian rhythm disturbances (*p* = 0.11), or parasomnias (*p* = 0.28) (Table 4).

**TABLE 4:** Effect of gender and disease severity on various psychiatric disorders and sleep quality.

		Insomnia	Psychiatric disorder	Circadian rhythm disorder	Movement disorder	Parasomnia	Poor sleep quality
Gender	Male (*n* = 53)	3 (6%)	5 (9%)	11 (21%)	2 (4%)	7 (13%)	39 (74%)
	Female (*n* = 116)	9 (8%)	25 (22%)	27 (23%)	16 (14%)	17 (15%)	86 (74%)
	*p* value	0.75	0.05	0.71	0.05	0.8	0.93

Disease severity	EDSS (0–3.0) (*n* = 108)	7 (7%)	20 (19%)	25 (23%)	8 (7%)	13 (12%)	78 (72%)
	EDSS 3.5–5.5 (*n* = 31)	3 (10%)	5 (16%)	10 (32%)	3 (10%)	4 (13%)	26 (84%)
	EDSS ≥ 6.0 (*n* = 30)	2 (7%)	5 (17%)	3 (10%)	7 (23%)	7 (23%)	21 (71%)
	*p* value	0.83	0.94	0.11	0.05	0.28	0.36

*Note:* EDSS = Expanded Disability Status Scale (higher scores indicate greater disability).

When multiple regression analysis was done for predictors of overall poor sleep quality, type of MS (OR = 1.886, 95% CI:0.295–12.064; *p* = 0.503), current EDSS score (OR = 1.02, 95% CI:0.364–2.868; *p* = 0.968), and current use of DMT (OR = 0.633, 95% CI:0.25–1.603; *p* = 0.335) did not show any clear relation.

## 4. Discussion

Poor sleep quality was very common in our cohort of pwMS, affecting about three‐fourths of patients. This prevalence exceeds that typically reported in prior MS studies (∼50%–67% of patients) and is markedly higher than rates in the general population (∼33%–45%) [1–11]. The use of a sensitive screening tool (PSQI) may account for this high rate. Notably, sleep‐related disorders in pwMS are often under‐recognized despite the significant impact on QoL, only a minority of patients spontaneously report sleep‐related issues to their healthcare providers [13]. Our findings emphasize the importance of proactively assessing sleep disturbances in pwMS, as patients may not volunteer this information.

Our results demonstrated a strong link between specific sleep‐related disorders and worse overall sleep quality. The mean global PSQI score in our sample was 7.8 (well above the cutoff of 5 for good sleep) [15], confirming that most patients had objectively poor sleep quality. Nearly all types of sleep‐related disorders were associated with significantly elevated PSQI scores. Patients who screened positive for insomnia, psychiatric disorders, circadian rhythm disorders, movement disorders, or parasomnia had roughly double the PSQI score of those without these conditions (*p* < 0.01 for each), indicating substantially worse subjective sleep quality. By contrast, those at high risk for sleep apnea did not significantly have raised PSQI. This finding is consistent with the observation that many pwMS experience difficulty initiating and maintaining sleep as well as daytime dysfunction, which are key components captured by the PSQI. In fact, every patient in our study who self‐identified as a “poor sleeper” was screened positive for insomnia, and the vast majority of those with poor sleep quality also reported co‐occurring psychiatric (97%) or circadian rhythm (90%) disorders. Even among those who considered themselves “good sleepers,” a considerable proportion had a potential sleep‐related disorder (e.g., 29% were at high risk for sleep apnea). This suggests that some pwMS may underappreciate milder sleep issues or tolerate them until those issues become more severe. This may also be one of the explanations of relatively infrequent finding of risk of insomnia in our cohort. The overall frequency of high risk for OSA was lower in our cohort compared to the previously published literature. The possible explanations may include only the risk assessment based on questionnaire, rather than objective testing for OSA. The estimate of risk of OSA increases with age, BMI > 35 kg/m^2^, male gender, and neck circumferences of more than 16 inches. pwMS in our cohort were mostly under 40 years of age, with BMI of < 30, mostly females, and had smaller neck circumference. All these factors may be another explanation of lower risk of OSA in this cohort.

We did not find a significant difference between men and women in overall sleep quality since both sexes had similarly high rates of poor sleep quality. This contrasts with some reports in the literature; for example, a recent meta‐analysis identified female sex as a risk factor for insomnia in MS [1]. In our cohort, however, sex did influence the pattern of sleep‐related disorders, although with borderline statistical significance: among women, risk of psychiatric sleep‐related disorders (22%) and circadian rhythm disorders (23%) were the most common, whereas in men, the risk of circadian rhythm disorders (21%) and parasomnias (13%) predominated. Gender therefore appeared to affect the type of sleep‐related disorder rather than the likelihood of poor sleep quality per se. It must be emphasized that the differences noted based on gender were borderline, and the findings may have been affected by the small number of pwMS in each group. The prominence of risk of circadian rhythm sleep–wake disorders and psychiatric disease–associated insomnia in our patients is notable, reflecting the multifactorial nature of sleep disruption in pwMS. Our findings are based on subjective reporting, and we did not have any objective measurement. We could not make definitive diagnosis of all the reported conditions and considered pwMS to be at risk of those conditions based on their response to questionnaires. These findings should be further studied by formal sleep studies to ascertain these differences.

We observed an interesting pattern of sleep disturbances across different disability levels (EDSS strata). Patients with moderate disability (EDSS 3.5–5.5) had the highest prevalence of poor sleep quality (84%), slightly higher than those with severe disability (EDSS ≥ 6, 70%) and those with mild disability (EDSS 0–3.0, 72%). This nonlinear trend suggests that sleep quality does not simply worsen in a stepwise fashion with neurological disability. Even patients with minimal disability can have significant sleep problems, as noted in other studies [14]. Due to the small number of pwMS in each group, these findings should be interpreted with caution, and may not be generalizable. Regarding specific sleep‐related disorder types, the moderate‐EDSS group showed the greatest proportions of risk of insomnia (10%) and circadian rhythm disturbances (32%), whereas the severely disabled group had more frequent risk of movement disorders (23%) and parasomnias (23%). Psychiatric sleep‐related disorders were relatively more commonly reported in mildly disabled patients (19%). These patterns imply that as MS progresses, the dominant contributors to sleep disruption may shift; mid‐stage disease perhaps increases risk of insomnia related to symptom burden or medication (e.g., corticosteroids) use, and late‐stage disease seeing more risk of nocturnal movement disorders and parasomnias due to cumulative neurodegeneration. However, our sample of severely disabled patients was small, so these differences should be interpreted with caution. Overall, the presence of sleep‐related disorders was widespread across all disability strata, reinforcing that clinicians should be vigilant for sleep issues even in patients with early or mild MS.

We did not find any significant associations between sleep quality and several other variables. Neither the MS subtype (relapsing‐remitting vs progressive) nor the use of DMTs showed a clear link to sleep quality in our cohort. These negative findings suggest that sleep‐related problems in pwMS may be driven more by disease‐intrinsic and psychosocial factors than by general risk profiles or specific MS subtypes. Consistent with this, recent studies in MS populations have not found a definitive impact of disease course or DMT use on sleep outcomes, emphasizing the need for larger multicenter studies to clarify these relationships [6–15].

The high frequency of subjective reporting of sleep‐related disorders in pwMS may have a significant impact on daily functioning, although we did not directly measure the impact of these sleep‐related disorders on pwMS through formal QoL measures. When formally interviewed for sleep‐related disorders in this study, the pwMS reported high frequency of sleep‐related disorders that highlights the need for routine screening of sleep‐related disorders in pwMS as part of standard care. Incorporating simple questionnaires like the PSQI or brief insomnia screens into regular MS clinic visits could help identify affected patients early. Prior literature has similarly emphasized that sleep issues should be routinely evaluated in MS care, especially since untreated sleep problems can exacerbate fatigue and other symptoms [16]. Managing sleep disorders in pwMS often requires a multidisciplinary approach. Neurologists, sleep specialists, and mental health professionals should collaborate to address the various contributors to poor sleep quality in these patients. Increasing physician awareness of the high risk of increased prevalence of sleep‐related disorders in pwMS (approximately three out of four patients in our study) is essential, as many patients might not volunteer sleep complaints during routine visits [13]. Proactively treating comorbid sleep‐related disorders has the potential to not only improve night‐time sleep quality but also reduce daytime fatigue, improve mood, and enhance overall QoL for pwMS [17].

This study has several limitations. First, it was a single‐center study with a relatively modest sample size and lacked a control group of healthy individuals, which limits generalizability. The cross‐sectional design also precludes causal inferences. Second, we relied on self‐reported questionnaires to identify sleep‐related disorders (including the PSQI and symptom‐based checklists) without objective confirmation by polysomnography or actigraphy. Self‐report measures can introduce bias and may overestimate or underestimate true sleep pathology. The positive risk for insomnia and high risk for OSA were relatively low in our cohort, which may be due to questionnaire‐based screening. If the patients had formal sleep studies, those numbers may have been different. Third, we did not fully account for all potential confounding comorbidities. For instance, chronic pain, spasticity, nocturnal urinary symptoms, and medications (such as corticosteroids or stimulants) could have influenced sleep quality in ways not captured by our analysis. These factors may partly explain the reported high prevalence of poor sleep quality and make it challenging to isolate the effect of MS itself.

Our findings suggest that regular screening of sleep quality be integrated into MS care, and further research is warranted to examine the impact of disease duration, coexisting risk factors, and comorbidities on sleep quality in pwMS. Future studies should include larger, multicenter cohorts (to improve generalizability) and ideally adopt longitudinal designs to track how sleep‐related disorders evolve with MS progression. Utilizing objective sleep assessments (e.g., polysomnography) alongside questionnaires would also help validate the findings and guide the development of targeted interventions [18].

## 5. Conclusions

In our cohort of pwMS, subjective reporting of sleep‐related disorders was common. High risk for insomnia, psychiatric disorders, and circadian rhythm disorders were associated with poor sleep quality. Certain reported disorders showed a trend toward female preponderance. Disease severity and use of DMTs did not affect sleep quality in our cohort.

pwMS should be inquired about sleep routinely, and optimal management should be offered to improve the QoL. Further, prospective studies in a larger number of patients are warranted to fully assess the impact and management of sleep‐related disorders in pwMS.

## Author Contributions

All authors have reviewed the manuscript.

## Funding

No funding was obtained for this research.

## Disclosure

This research was presented as a poster presentation in the 22^nd^ Annual Department of Medicine Research Day, King Abdulaziz Medical City, MNGHA, Riyadh, KSA on March 17, 2022.

This research was published as abstract in Journal of Neurological Sciences for the 25^th^ World Congress of Neurology, Rome, Italy, October 2021. DOI: 10.1016/j.jns.2021.118181.

All the co‐authors have read the manuscript and approved it for final submission. All authors have agreed for the manuscript publication. This manuscript was not previously published in any journal.

## Ethics Statement

The study was approved by the institutional review board of King Abdullah International Medical Research Center (KAIMRC), number: RC19/099/R.

## Consent

Informed consent was obtained from the participants on the IRB‐approved consent form.

## Conflicts of Interest

Dr. Yaser Al Malik has received honoraria from Biogen, Novartis, Roche, Merck, Sanofi Genzyme, and Janssen.

The other authors declare no conflicts of interest.

## Data Availability

The data that support the findings of this study are available from the corresponding author upon reasonable request.
